# Supplementary material for: Pyruvate Dehydrogenase Kinase 1 inhibition mediated oxidative phosphorylation enhancement in cartilage promotes osteoarthritis progression
Source: BMC Musculoskelet Disord. 2023 Jul 20;24:597. doi: 10.1186/s12891-023-06585-6 (PMC10357736; doi:10.1186/s12891-023-06585-6)
Supplement: Supplementary file 1 — Additional file 1. [file 12891_2023_6585_MOESM1_ESM.zip › BP.Gsea.1653623667859/GOBP_ANGIOGENESIS_INVOLVED_IN_WOUND_HEALING.html]

Details for gene set GOBP\_ANGIOGENESIS\_INVOLVED\_IN\_WOUND\_HEALING[GSEA]

|  || Dataset | 55457\_collapsed\_to\_symbols.cls.cls #OA\_versus\_normal.cls.cls #OA\_versus\_normal\_repos |
| Phenotype | cls.cls#OA\_versus\_normal\_repos |
| Upregulated in class | normal |
| GeneSet | GOBP\_ANGIOGENESIS\_INVOLVED\_IN\_WOUND\_HEALING |
| Enrichment Score (ES) | -0.5478432 |
| Normalized Enrichment Score (NES) | -1.7490412 |
| Nominal p-value | 0.010471204 |
| FDR q-value | 0.11762225 |
| FWER p-Value | 0.999 |
Table: GSEA Results Summary

  

Fig 1: Enrichment plot: GOBP\_ANGIOGENESIS\_INVOLVED\_IN\_WOUND\_HEALING      
 Profile of the Running ES Score & Positions of GeneSet Members on the Rank Ordered List

  

| SYMBOL | TITLE | RANK IN GENE LIST | RANK METRIC SCORE | RUNNING ES | CORE ENRICHMENT || 1 | TNF | tumor necrosis factor [Source:HGNC Symbol;Acc:HGNC:11892] | 2098 | 0.350 | -0.1226 | No |
| 2 | PIK3CB | "phosphatidylinositol-4,5-bisphosphate 3-kinase catalytic subunit beta [Source:HGNC Symbol;Acc:HGNC:8976]" | 2186 | 0.340 | -0.0902 | No |
| 3 | HPSE | heparanase [Source:HGNC Symbol;Acc:HGNC:5164] | 2360 | 0.319 | -0.0668 | No |
| 4 | NDNF | neuron derived neurotrophic factor [Source:HGNC Symbol;Acc:HGNC:26256] | 2988 | 0.251 | -0.0866 | No |
| 5 | DAG1 | dystroglycan 1 [Source:HGNC Symbol;Acc:HGNC:2666] | 3203 | 0.230 | -0.0767 | No |
| 6 | ALOX5 | arachidonate 5-lipoxygenase [Source:HGNC Symbol;Acc:HGNC:435] | 3776 | 0.179 | -0.1005 | No |
| 7 | CD34 | CD34 molecule [Source:HGNC Symbol;Acc:HGNC:1662] | 4341 | 0.125 | -0.1299 | No |
| 8 | CX3CL1 | C-X3-C motif chemokine ligand 1 [Source:HGNC Symbol;Acc:HGNC:10647] | 5764 | 0.011 | -0.2390 | No |
| 9 | CXCR4 | C-X-C motif chemokine receptor 4 [Source:HGNC Symbol;Acc:HGNC:2561] | 6382 | -0.034 | -0.2831 | No |
| 10 | PRCP | prolylcarboxypeptidase [Source:HGNC Symbol;Acc:HGNC:9344] | 6725 | -0.057 | -0.3030 | No |
| 11 | ADIPOR2 | adiponectin receptor 2 [Source:HGNC Symbol;Acc:HGNC:24041] | 6729 | -0.058 | -0.2966 | No |
| 12 | MCAM | melanoma cell adhesion molecule [Source:HGNC Symbol;Acc:HGNC:6934] | 8769 | -0.214 | -0.4303 | No |
| 13 | SERPINE1 | serpin family E member 1 [Source:HGNC Symbol;Acc:HGNC:8583] | 10284 | -0.349 | -0.5076 | Yes |
| 14 | ITGB3 | integrin subunit beta 3 [Source:HGNC Symbol;Acc:HGNC:6156] | 10310 | -0.352 | -0.4690 | Yes |
| 15 | GATA2 | GATA binding protein 2 [Source:HGNC Symbol;Acc:HGNC:4171] | 11058 | -0.439 | -0.4764 | Yes |
| 16 | KDR | kinase insert domain receptor [Source:HGNC Symbol;Acc:HGNC:6307] | 11454 | -0.496 | -0.4499 | Yes |
| 17 | B4GALT1 | "beta-1,4-galactosyltransferase 1 [Source:HGNC Symbol;Acc:HGNC:924]" | 11530 | -0.505 | -0.3975 | Yes |
| 18 | SLC12A2 | solute carrier family 12 member 2 [Source:HGNC Symbol;Acc:HGNC:10911] | 11744 | -0.540 | -0.3518 | Yes |
| 19 | FOXC2 | forkhead box C2 [Source:HGNC Symbol;Acc:HGNC:3801] | 12218 | -0.650 | -0.3136 | Yes |
| 20 | SRF | serum response factor [Source:HGNC Symbol;Acc:HGNC:11291] | 12549 | -0.773 | -0.2501 | Yes |
| 21 | GPR4 | G protein-coupled receptor 4 [Source:HGNC Symbol;Acc:HGNC:4497] | 12561 | -0.778 | -0.1613 | Yes |
| 22 | VEGFA | vascular endothelial growth factor A [Source:HGNC Symbol;Acc:HGNC:12680] | 12629 | -0.813 | -0.0728 | Yes |
| 23 | TNFAIP3 | TNF alpha induced protein 3 [Source:HGNC Symbol;Acc:HGNC:11896] | 12631 | -0.814 | 0.0209 | Yes |
Table: GSEA details [plain text format]

  

Fig 2: GOBP\_ANGIOGENESIS\_INVOLVED\_IN\_WOUND\_HEALING      
 Blue-Pink O' Gram in the Space of the Analyzed GeneSet

  

Fig 3: GOBP\_ANGIOGENESIS\_INVOLVED\_IN\_WOUND\_HEALING: Random ES distribution      
 Gene set null distribution of ES for **GOBP\_ANGIOGENESIS\_INVOLVED\_IN\_WOUND\_HEALING**
